# Laudanum in the Delirium Occurring in Dothinenteritis

**Published:** 1846-04

**Authors:** 


*Laudanum in the Delirium occurring in the last stage of Dothinenteritis.*—Dr. Morand, of Tours, is of opinion that great advantage may be derived from certain preparations of opium in the treatment of the delirium which supervenes in the last stage of the dothinenteritis. He relates a case of a girl 11 years of age, who, on the 32d day of an attack of dothinenteritis, seemed in the most hopeless condition. She had continued delirium, and screamed without cessation. A potion containing fifteen drops of laudunum was ordered to be given every hour, which quieted her cries, procured sleep, and in 18 hours the delirium ceased. The potion was afterwards continued for a few days to prevent a return of the delirium, and with a nourishing regimen the patient entirely recovered.—*Jour. de Méd.*—*Ibid.*

